# Quantum Features of Macroscopic Fields: Entropy and Dynamics

**DOI:** 10.3390/e21070705

**Published:** 2019-07-18

**Authors:** Robert Alicki

**Affiliations:** International Centre for Theory of Quantum Technologies (ICTQT), University of Gdańsk, 80-308 Gdańsk, Poland; fizra@univ.gda.pl; Tel.: +48-503-066-248

**Keywords:** classical field theory, quantum open systems, von Neumann entropy, Mueller Jones calculi

## Abstract

Macroscopic fields such as electromagnetic, magnetohydrodynamic, acoustic or gravitational waves are usually described by classical wave equations with possible additional damping terms and coherent sources. The aim of this paper is to develop a complete macroscopic formalism including random/thermal sources, dissipation and random scattering of waves by environment. The proposed reduced state of the field combines averaged field with the two-point correlation function called single-particle density matrix. The evolution equation for the reduced state of the field is obtained by reduction of the generalized quasi-free dynamical semigroups describing irreversible evolution of bosonic quantum field and the definition of entropy for the reduced state of the field follows from the von Neumann entropy of quantum field states. The presented formalism can be applied, for example, to superradiance phenomena and allows unifying the Mueller and Jones calculi in polarization optics.

## 1. Introduction

It is generally believed that low frequency waves appearing in the macroscopic world such as various types of mechanical waves including acoustic ones, magnetohydrodynamic, radio-frequency electromagnetic or gravitational waves can be successfully described using classical wave equations with external sources [1]. This is certainly true for coherent deterministic sources while the case of thermal and generally random sources is much more problematic. It is not obvious how to incorporate classical waves as a part of thermodynamical system where exchange of heat, entropy production and generation of work should be taken into account. A simple addition of damping terms to wave equations is not sufficient. In particular, the question of defining entropy for macroscopic fields and appropriate formulation of the Second Law is still an open problem. On the other hand, it is clear that the ultimate fundamental description of physical systems should be given by the quantum theory. However, the full formalism of the quantum field theory is too complicated and not convenient for practical applications. In most cases, the relevant observables (energy, mass, momentum, angular momentum, and polarization) are given by quadratic forms of fields and the linear approximation for dynamical equations is sufficient or can be easily amended by self-consistent non-linear corrections. This is similar to the case of classical gas where the description in terms of particle density in the single-particle phase-space and the dynamics given by (linear) Boltzmann equation is more useful than the complete N-body formalism.

The aim of this paper is to propose a novel approach to macroscopic description of fields based on the notions of single-particle density matrix and averaged field (called here reduced state of the field). This formalism allows computing all additive quantities including the entropy of the macroscopic field also introduced here. The definition of entropy involves maximum entropy principle applied to the von Neumann entropy of quantum field. A new family of quantum Markovian master equations is proposed, generalizing the previous framework of quasi-free quantum dynamical maps and semigroups [2,3,4,5]. It includes processes of linear damping/pumping and random scattering of waves by an environment. The exact reduction procedure is performed leading to kinetic equations for the reduced states of the field. For the case of purely reversible processes, these kinetic equations reduce further to standard wave equations.

To illustrate in the simplest way the quantum features of classical fields, we begin with the discussion of light polarization in terms of Stokes parameters and its quantum-mechanical interpretation. Then, we present a complete description of a quantum field in terms of modes and its *First Quantization* interpretation, where the classical field is treated as a (not normalized) quantum wave function of the corresponding (quasi)particle. The further step called *Second Quantization* allows describing irreversible processes in terms of Markovian Master Equations for density matrices acting of the corresponding bosonic Fock space. In the final step, we develop the *reduced state of the field* formalism and apply it to the case of thermal sources and polarization optics.

## 2. Jones and Mueller Formalism for Light Polarization

In optics, polarized light can be described using the Jones calculus, while the partially polarized one is treated using Mueller calculus [6].

Consider first a monochromatic plane wave of light propagating along Axis 3 in a Cartesian frame with the basis ${\hat{\epsilon }}_{k}$, k=1,2,3. The (pure) state of polarization is specified by the complex amplitudes of the electric field, $E_{1}$ and $E_{2}$, in a basis $\left({\hat{\epsilon }}_{1},{\hat{\epsilon }}_{2}\right)$ The pair $\left(E_{1},E_{2}\right)$ is called the *Jones vector* and contains both amplitudes and phases of two orthogonal components of the wave electric field. The relevant parameter is the normalization of the Jones vector

(1) $$s_{0}=|E_{1}{|}^{2}+{\left|E_{2}\right|}^{2}$$

which is proportional to the energy density of the wave, hence also to the intensity of light beam *I*, and, using quantum picture of light, to the averaged photon number *N*. All those interpretations of $s_{0}$ can be useful in applications. With a given Jones vector, we can associate a 2×2 complex-valued matrix with matrix elements $E_{k}{\bar{E}}_{\ell }$. The main idea of Stokes was to average those matrix elements with respect to fluctuations due either to slow fluctuations in time or the contribution to the light beam from different uncorrelated sources. Such a *Stokes matrix* $\hat{S}$ can be decomposed with respect to Pauli matrices $\left\{{\hat{\sigma }}_{k},k=1,2,3\right\}$ as

(2) $$\hat{S}\equiv \left[\langle E_{k}{\bar{E}}_{\ell }\rangle \right]=\frac{1}{2}\sum_{\mu =0}^{3}s_{\mu }{\hat{\sigma }}_{\mu }$$

with unite matrix ${\hat{\sigma }}_{0}\equiv 1$. The real parameters $s_{\mu }$ are called *Stokes parameters* and form a four-dimensional *Stokes vector* $s\equiv (s_{0},\vec{s})$. As the Stokes matrix $\hat{S}$ is positively defined, the Stokes vector satisfies inequality

(3) $$s_{0}^{2}\geq s_{1}^{2}+s_{2}^{2}+s_{3}^{2}.$$

Stokes anticipated that those parameters provide a *complete description of polarization state* of the monochromatic light beam. This assumption lies behind the Mueller calculus, which describes the action of any *linear* optical device by 4×4 Mueller matrix $M=[M_{\mu \nu }]$ acting on the input Stokes vector S and yielding the output one $S^{\prime }$

(4) $$s_{\mu }^{\prime }=\sum_{\nu =0}^{s}M_{\mu \nu }\;s_{\nu }.$$

Although the Stokes matrix/vector is constructed from classical correlations between components of the classical electric fields, the above completeness assumption is not consistent with the classical probabilistic scheme. Namely, by treating polarization as a classical dynamical variable, each fully polarized state of light satisfying the equality in Equation (3) with a fixed intensity $s_{0}$ corresponds to a pure state of the system. The set of all such pure states form the so-called *Poincare sphere*. Therefore, a classical mixed state of polarization corresponding to a partially or completely polarized monochromatic light beam with a given intensity should be described by a probability measure on the Poincare sphere. Hence, the set of all mixed states is an infinite-dimensional simplex of all probability measures on the Poincare sphere generated by extreme points—the Dirac measures concentrated on all points of the sphere. On the other hand, in Stokes formalism, any mixed state of polarization is given by the three-dimensional vector with the length smaller or equal to $s_{0}$. This is completely equivalent to the description of quantum mixed states for the two-level systems with Poincare sphere replaced by *Bloch sphere*. Therefore, one can say that Stokes was the first who discovered the quantum nature of light, but similarly to Columbus was not aware of the meaning of his discovery [7].

The equivalence mentioned above sheds a new light on the Jones and Mueller calculi, which are useful tools in polarization optics. Namely, using the well-known results from the quantum theory of open systems [5,8,9], we can assume that any Mueller matrix corresponds to a completely positive map Φ such that Equation (4) is equivalent to

(5) $${\hat{S}}^{\prime }=\Phi \left(\hat{S}\right)=\sum_{\alpha }{\hat{V}}_{\alpha }\hat{S}{\hat{V}}_{\alpha }^{†}$$

where the 2×2 complex matrices ${\hat{V}}_{\alpha }$ are not uniquely defined, but one can always find the representation of Φ with at most four such matrices. The special case is a map Φ given by a single matrix $\hat{V}$, which means that each completely polarized state is transformed into another completely polarized one, albeit with different intensity. Therefore, one can restrict the description to map $\hat{V}$ acting on Jones vectors, which is the essence of *Jones calculus*.

The general Mueller map is completely positive but not trace preserving as $\mathrm{Tr}\left(\hat{S}\right)=s_{0}$ and the intensity can change under the action of linear optics device. In principle, this theory describes not only absorption of light by the passive devices but also its amplification by active medium.

The natural question, related to the thermodynamic properties of polarized light, is the definition of entropy for a monochromatic light beam with a given polarization state described by $\hat{S}$. This question is discussed in Section 4 after generalization of Stokes formalism to other degrees of freedom of the macroscopic field.

## 3. First- and Second-Quantization of Classical Field

In this paper, we restrict ourselves to the classical field occupying a finite volume and hence described by the set of complex modes $f_{k}\left(x\right)$, where *x* is the position vector and *k* denotes a discrete index. The modes evolve in time according to the formula

(6) $$f_{k}\left(x;t\right)=e^{-i\omega_{k}t}f_{k}\left(x\right)$$

and the arbitrary solution of the corresponding wave equation can be represented by linear combination of modes.

In the picture of *first-quantization* modes, $\left\{f_{k}\right\}$ correspond to (generally not normalized) energy eigenstates of the single-particle Hamiltonian $\hat{h}$ describing a single (quasi)particle associated with the field (e.g., photon, graviton, phonon, magnon, etc.). The quantum single-particle Hilbert space H is spanned by those modes, with a proper normalization such that:

(1) $\left\{f_{k}\right\}$ form an orthonormal basis in H; and
(2) the classical energy of the field mode $f_{k}$ equals $\hbar \omega_{k}$.

From now on, we identify the classical field configuration represented by the linear superposition of modes with the corresponding vector in the Hilbert space of the first quantization. Therefore, the only mathematical difference between classical field and the first-quantization interpretation is the chosen normalization. In the first case, we normalize field to the given energy or intensity, while in the second case to one treating classical field as a wave function of a single particle.

The *second-quantization* formalism describes the quantum field in terms of bosonic Fock space $H_{F}$ with a set of annihilation and creation operators $\left\{{\hat{a}}_{k},{\hat{a}}_{k}^{†}\right\}$ corresponding to the modes $\left\{f_{k}\right\}$. They satisfy the canonical commutation relations

(7) $$\left[{\hat{a}}_{k},{\hat{a}}_{k^{\prime }}\right]=\left[{\hat{a}}_{k}^{†},{\hat{a}}_{k^{\prime }}^{†}\right]=0,\;\left[{\hat{a}}_{k},{\hat{a}}_{k^{\prime }}^{†}\right]=\delta_{kk^{\prime }}.$$

The Fock space is spanned by the vectors obtained by application of all monomials in creation operators acting on the *vacuum state*

(8) $${\left({\hat{a}}_{k_{1}}\right)}^{n_{1}}{\left({\hat{a}}_{k_{2}}^{†}\right)}^{n_{2}}\cdots {\left({\hat{a}}_{k_{m}}^{†}\right)}^{n_{m}}|0\rangle .$$

For any vector $|\alpha \rangle =\sum_{k}\alpha_{k}|k\rangle$ in the single-particle Hilbert space there exists a normalized vector (pure state) $|\alpha_{F}\rangle$ in the Fock space, called *coherent state*, which is a joint eigenvector of all annihilation operators

(9) $${\hat{a}}_{k}|\alpha_{F}\rangle =\alpha_{k}|\alpha_{F}\rangle .$$

The coherent state can be explicitly written as

(10) $$|\alpha_{F}\rangle =\sum_{n=0}^{\infty }\frac{1}{\sqrt{n!}}{\left({\hat{a}}^{†}\left[\alpha \right]\right)}^{n}|0\rangle \;\mathrm{where}\;{\hat{a}}^{†}\left[\alpha \right]\equiv \sum_{k}\alpha_{k}{\hat{a}}_{k}^{†},$$

or, introducing the Weyl unitary operators $\hat{W}\left[\alpha \right]$ on the Fock space, as

(11) $$|\alpha_{F}\rangle =\hat{W}\left[\alpha \right]|0\rangle ,\;\mathrm{where}\;\hat{W}\left[\alpha \right]=e^{\left(\hat{a}\left[\alpha \right]-{\hat{a}}^{†}\left[\alpha \right]\right)}.$$

The coherent state $|\alpha_{F}\rangle$ can be treated as the quantum analog of the classical field |α〉.

In the following, we restrict ourselves to two classes of operators acting on the Fock space obtained by two different *lifting procedures* applied to operators acting on the single-particle Hilbert space.

The single particle observable $\hat{b}$ expressed in the basis $\{|k\rangle \equiv |f_{k}\rangle \}$ as

(12) $$\hat{b}=\sum_{k,k^{\prime }}b_{kk^{\prime }}|k\rangle \langle k^{\prime }|$$

produces an *additive observable* on the Fock space $H_{F}$ of the form

(13) $$\hat{B}=\sum_{k,k^{\prime }}b_{kk^{\prime }}{\hat{a}}_{k}^{†}{\hat{a}}_{k^{\prime }}.$$

In particular, for the Hamiltonian, we have

(14) $$\hat{h}=\hbar \sum_{k}\omega_{k}|k\rangle \langle k|,\;\hat{H}=\hbar \sum_{k}\omega_{k}{\hat{a}}_{k}^{†}{\hat{a}}_{k}\;,$$

and the number operator is denoted by $\hat{N}=\sum_{k}{\hat{a}}_{k}^{†}{\hat{a}}_{k}$.

Any unitary operator $\hat{u}$ acting on the single-particle Hilbert space extends to the Fock space *multiplicative unitary* $\hat{U}$, which can be defined in two equivalent ways

(15) $$\hat{u}=e^{i\hat{b}},\;\hat{U}=e^{i\hat{B}},$$

or

(16) $$\hat{U}\hat{C}{\hat{U}}^{†}=\hat{D},\;\mathrm{where}\;\hat{d}=\hat{u}\hat{c}{\hat{u}}^{†}.$$

The action of $\hat{U}$ can be also defined in terms of coherent states or Weyl unitaries

(17) $$\hat{U}|\alpha_{F}{\rangle =|\left(u\alpha \right)}_{F}\rangle ,\;\hat{U}\hat{W}\left[\alpha \right]{\hat{U}}^{†}=\hat{W}\left[u\alpha \right]$$

where $|u\alpha \rangle \equiv \hat{u}|\alpha \rangle$.

For the non-interacting field with dynamics governed by linear field equations with possible external classical and coherent sources, the fundamental measurable quantities, such as energy, momentum and angular momentum are additive observables. Therefore, instead of the full density matrix ${\hat{\rho }}_{F}$ acting of the Fock space $H_{F}$, we can use the *single-particle density matrix* $\hat{\rho }$ acting on the single-particle Hilbert space H. The reduction map ${\hat{\rho }}_{F}\Rightarrow \hat{\rho }$ satisfies the following conditions

(18) $$\mathrm{Tr}\left({\hat{\rho }}_{F}\hat{B}\right)=\mathrm{tr}\left(\hat{\rho }\hat{b}\right).$$

and

(19) $$\hat{U}{\hat{\rho }}_{F}{\hat{U}}^{†}\Rightarrow \hat{u}\hat{\rho }{\hat{u}}^{†}.$$

Here, the trace Tr always refers to the Fock space $H_{F}$ and tr to the H.

The explicit form of a single-particle density matrix is given by

(20) $$\hat{\rho }=\sum_{k,k^{\prime }}\mathrm{Tr}\left({\hat{\rho }}_{F}{\hat{a}}_{k^{\prime }}^{†}{\hat{a}}_{k}\right)|k\rangle \langle k^{\prime }|,$$

which can be treated as the generalization of the idea of Stokes matrix to other degrees of freedom, beyond polarization. Notice that the single-particle density matrix is normalized to the averaged number of particles,

(21) $$\mathrm{tr}\hat{\rho }=N=\mathrm{Tr}\left({\hat{\rho }}_{F}\hat{N}\right).$$

The additional information about the phases of the field is contained in the *averaged field* |α〉, which is a vector in the single-particle Hilbert space H defined as

(22) $$|\alpha \rangle =\sum_{k}\mathrm{Tr}\left({\hat{\rho }}_{F}{\hat{a}}_{k}\right)|k\rangle ,$$

generalizing the idea of Jones vector.

The definitions in Equations (13) and (20) imply that the *correlation matrix* given by the formula

(23) $${\hat{\rho }}^{\alpha }=\hat{\rho }-\left|\alpha \rangle \langle \alpha \right|\geq 0$$

is a positive operator on the single-particle Hilbert space.

The *reduced description* in terms of the pair $\left(\hat{\rho },|\alpha \rangle \right)$ called *reduced state of the field* contains sufficient information about the most important properties of the macroscopic field interacting with environment. The reduced state of field is called *pure* if $\hat{\rho }=\left|\alpha \rangle \langle \alpha \right|$ or, equivalently ${\hat{\rho }}^{\alpha }=0$. One can easily prove that the reduced state of the field is pure if and only if the original state of the quantum field is coherent.

## 4. Quantum Entropy of Macroscopic Field

In phenomenological thermodynamics of equilibrium systems, entropy is a function of a macroscopic state, which is characterized by a small number of controlled external parameters and temperature. Already in this case we have a certain freedom in selecting those external parameters related to our ability of controlling the system. The situation is more complicated for non-equilibrium systems where, typically, thermodynamic parameters including temperature become position-dependent and their choice is determined by the relevant time-scales of local equilibration processes.

A similar problem appears when the notion of entropy is discussed within classical or quantum statistical mechanics. The proper choice of the definition depends on the selected level of complexity of our theoretical framework. This level is determined by the set of accessible observables of the system, which can be measured and/or controlled. Again, this choice is also related to relevant time-scales. Such “subjectivity” in the definition of entropy does not lead to any inconsistencies. Namely, the basic thermodynamical quantity with direct physical interpretation depending on entropy is the *free energy* F=U−TS (where *U* is the internal energy, *T* is the temperature, and *S* is the entropy), which determines the amount of work extractable from the system. Obviously, both extractable work and entropy depend on the available means of control over the system.

To illustrate the problem of selection of complexity level, we consider the classical gas of *N* identical particles in a finite volume.The complete microscopical and statistical description of the state of such system is given by the *N*-particle probability distribution $p_{N}\left({\vec{r}}_{1},{\vec{p}}_{1},\cdots ,{\vec{r}}_{N},{\vec{p}}_{N}\right)$, which is symmetric with respect to permutations.

The natural choice for the entropy of such state is the Gibbs expression

(24) $$S^{G}\left(p_{N}\right)=-k_{B}\int \cdots \int p_{N}\left({\vec{r}}_{1},{\vec{p}}_{1},\cdots ,{\vec{r}}_{N},{\vec{p}}_{N}\right)\log p_{N}\left({\vec{r}}_{1},{\vec{p}}_{1},\cdots ,{\vec{r}}_{N},{\vec{p}}_{N}\right)\;d^{3}{\vec{r}}_{1}\;d^{3}{\vec{p}}_{1},\cdots ,d^{3}{\vec{r}}_{N}\;d^{3}{\vec{p}}_{N}.$$

However, the typical means of control over the gas are based on additive observables which does not involve correlations between individual particles. Therefore, for practical purposes, the statistical description of gas in terms of marginal single-particle probability distribution $p(\vec{r},\vec{p})$ is sufficient, at least for the low density regime. The standard definition of entropy in this case is the Boltzmann one, used in his description of gas dynamics

(25) $$S^{B}=-k_{B}N\int p\left(\vec{r},\vec{p}\right)\log p\left(\vec{r},\vec{p}\right)\;d^{3}\vec{r}\;d^{3}\vec{p},$$

which coincides with Equation (24) in the case of product probability distribution $p_{N}={⊓}_{j=1}^{N}p\left({\vec{r}}_{j},{\vec{p}}_{j}\right)$.

Among the *N*-particle probability distributions with the same marginal $p(\vec{r},\vec{p})$, the product distribution maximizes the Gibbs entropy. Therefore, the Boltzmann choice in Equation (25) can be treated as the instance of the *Maximum Entropy Principle* applied to the single-particle reduced description [10].

We follow the analogous reasoning for the case of macroscopic field described in terms of the reduced state of the field $\left(\hat{\rho },|\alpha \rangle \right)$. Here, in contrast to the low density gas where two-particle correlations can be neglected, for the boson gas, strong correlations caused by quantum statistics and fully accounted by the second-quantization formalism are present. They dominate over possible interactions between (quasi)particles, which are anyway neglected in the linear (linearized) theory. One should also remember that, paradoxically, macroscopic fields correspond to the highly non-classical regime in the sense of the theory of quantum gases. For example, the thermal equilibrium population number for the 1 kHz acoustic mode at room temperature is of the order $10^{10}$, while classical theory of gases is valid for population numbers much smaller than one.

Consider first the *quasi-free state* on the Fock space, generated by the additive observable $\hat{R}$ corresponding to the single-particle observable $\hat{b}$, which has form

(26) $${\hat{\rho }}_{F}^{\prime }=\frac{e^{-\hat{R}}}{\mathrm{Tr}e^{-\hat{R}}}.$$

One can easily compute the reduced state of the field corresponding to the state in Equation (26) obtaining

(27) $${\hat{\rho }}^{\prime }=\frac{1}{e^{\hat{r}}-1},\;|\alpha^{\prime }\rangle =0,$$

and its von Neumann entropy

(28) $$S_{vN}\left[{\hat{\rho }}_{F}^{\prime }\right]=-k_{B}\mathrm{Tr}\left({\hat{\rho }}_{F}^{\prime }\log{\hat{\rho }}_{F}^{\prime }\right)=k_{B}\mathrm{tr}\left(\left({\hat{\rho }}^{\prime }+1\right)\log\left({\hat{\rho }}^{\prime }+1\right)-{\hat{\rho }}^{\prime }\log{\hat{\rho }}^{\prime }\right).$$

To include also macroscopic coherence, one can apply the Weyl unitary transformation to produce the new state

(29) $${\hat{\rho }}_{F}=\hat{W}\left[\alpha \right]{\hat{\rho }}_{F}^{\prime }{\hat{W}}^{†}\left[\alpha \right].$$

The reduced state of the field corresponding to the state in Equation (29) is now $\left(\hat{\rho },|\alpha \rangle \right)$ with

(30) $$\hat{\rho }={\hat{\rho }}^{\prime }+|\alpha \rangle \langle \alpha |.$$

It is not difficult to check that ${\hat{\rho }}_{F}$ of the form in Equation (29) maximizes von Neumann entropy among all states on the Fock space with the given ($\hat{\rho },|\alpha \rangle$). Therefore, we can define the entropy of the reduced state of the field by the von Neumann entropy $S_{vN}\left[{\hat{\rho }}_{F}\right]=S_{vN}\left[{\hat{\rho }}_{F}^{\prime }\right]$, which yields the expression depending on the correlation matrix ${\hat{\rho }}^{\alpha }=\hat{\rho }-\left|\alpha \rangle \langle \alpha \right|={\hat{\rho }}^{\prime }$

(31) $$S[\hat{\rho };|\alpha \rangle ]=k_{B}\mathrm{tr}\left(\left({\hat{\rho }}^{\alpha }+1\right)\log\left({\hat{\rho }}^{\alpha }+1\right)-{\hat{\rho }}^{\alpha }\log{\hat{\rho }}^{\alpha }\right).$$

This entropy satisfies the natural conditions $S[\hat{\rho };|\alpha \rangle ]\geq 0$ and $S[\hat{\rho };|\alpha \rangle ]=0$ if and only if $\hat{\rho }=\left|\alpha \rangle \langle \alpha \right|$, i.e., the reduced state of the field is pure.

The fact that only coherent states of the quantum field produce zero entropy reduced states of the field and all other pure states on the Fock space do not illustrates the dependence of the notion of entropy on complexity of description, which is determined by the assumed level of control. It might sound counterintuitive that a pure state on the Fock space, e.g., N-photon state, leads to non-zero macroscopic entropy, while a coherent state with the same averaged photon number gives zero entropy. However, from the point of view of joint measurement of averaged photon number and phase of the field, N-photon state has no definite phase and hence contains less information with respect to the selected measurement/control scheme.

## 5. Generalized Quasi-Free Dynamics

The so-called quasi-free dynamical semigroups are completely positive trace preserving semigroups of dynamical maps acting on Fock space density matrices, which from the physical standpoint describe particle decay and production processes in the approximation of independent particles. In the following, we introduce a more general class of such dynamical semigroups, which include, additionally, coherent classical source and individual and random scattering by the environment. It is assumed that a single scattering process is unitary, hence does not produce a persistent entanglement with environment. The master equation satisfying the assumptions of above takes the form ({·,·} denotes anticommutator)

(32) $$\begin{array}{cc} \frac{d}{dt}{\hat{\rho }}_{F} & =-i\hbar \sum_{k}\omega_{k}\left[{\hat{a}}_{k}^{†}{\hat{a}}_{k},{\hat{\rho }}_{F}\right]+\sum_{k}\left[\left(\xi_{k}{\hat{a}}_{k}^{†}-{\bar{\xi }}_{k}{\hat{a}}_{k}\right),{\hat{\rho }}_{F}\right]\\ & +\sum_{k,k^{\prime }}\Gamma_{↓}^{kk^{\prime }}({\hat{a}}_{k}{\hat{\rho }}_{F}{\hat{a}}_{k^{\prime }}^{†}-\frac{1}{2}\left\{{\hat{a}}_{k^{\prime }}^{†}{\hat{a}}_{k},{\hat{\rho }}_{F}\right\})\\ & +\sum_{k,k^{\prime }}\Gamma_{↑}^{kk^{\prime }}({\hat{a}}_{k}^{†}{\hat{\rho }}_{F}{\hat{a}}_{k^{\prime }}-\frac{1}{2}\left\{{\hat{a}}_{k^{\prime }}{\hat{a}}_{k}^{†},{\hat{\rho }}_{F}\right\})\\ & +\int \mu \left(du\right)(\hat{U}{\hat{\rho }}_{F}{\hat{U}}^{†}-{\hat{\rho }}_{F}). \end{array}$$

In the formula of above, the complex amplitudes $\xi_{k}$ describe the coherent source of field, and the positive matrices $\left[\Gamma_{↓}^{kk^{\prime }}\right]$ and $\left[\Gamma_{↑}^{kk^{\prime }}\right]$ contain particle annihilation and production rates for random sources. Those rates are expressed as the eigenvalues of $\left[\Gamma_{↓}^{kk^{\prime }}\right]$ and $\left[\Gamma_{↑}^{kk^{\prime }}\right]$, respectively. The last term describes random scattering processes parameterized by the positive measure μ(du), or more generally, positive distribution defined on the group of all unitaries acting on H. In particular, when the Poisson process of random scattering tends to its diffusion limit, one obtains the *double commutator* terms $-[\hat{Q},\left[\hat{Q},{\hat{\rho }}_{F}\right]]$, with an additive observable $\hat{Q}$, often used to describe pure decoherence.

Equation (32) possesses the standard Gorini–Kossakowski–Lindblad–Sudarshan form [8,9,11,12] and hence its solution is given by the completely positive trace-preserving dynamical semigroup.

By direct calculation, one can obtain from Equation (32) the closed evolution equation for the reduced description of the field in terms of the reduced state of the field $\left(\hat{\rho },|\alpha \rangle \right)$. Introducing the single-particle positive operators representing damping and pumping

(33) $$\hat{\gamma_{↓}}=\sum_{k,k^{\prime }}\Gamma_{↓}^{kk^{\prime }}|k\rangle \langle k^{\prime }|,\;\hat{\gamma_{↑}}=\sum_{k,k^{\prime }}\Gamma_{↑}^{kk^{\prime }}|k\rangle \langle k^{\prime }|,$$

and the single-particle vector describing coherent source

(34) $$|\xi \rangle =\sum_{k}\xi_{k}|k\rangle ,$$

one can write the equations of motion in the form of two coupled equations, which are hereafter called *reduced kinetic equations* for reduced states of the field.

(35) $$\begin{array}{cc} \frac{d}{dt}\hat{\rho } & =-\frac{i}{\hbar }\left[\hat{h},\hat{\rho }\right]+(|\xi \rangle \langle \alpha |+|\alpha \rangle \langle \xi |)\\ & +\left\{\left({\hat{\gamma }}_{↑}-{\hat{\gamma }}_{↓}\right),\hat{\rho }\right\}+{\hat{\gamma }}_{↑}\\ & +\int \mu \left(du\right)(\hat{u}\hat{\rho }{\hat{u}}^{†}-\hat{\rho }). \end{array}$$

(36) $$\begin{array}{cc} \frac{d}{dt}|\alpha \rangle & =-\frac{i}{\hbar }\hat{h}|\alpha \rangle +\frac{1}{2}\left({\hat{\gamma }}_{↑}-{\hat{\gamma }}_{↓}\right)|\alpha \rangle +|\xi \rangle \\ & +\int \mu \left(du\right)\left(\hat{u}-1)\right)|\alpha \rangle . \end{array}$$

The reduced kinetic equations (Equations (35) and (36)) are exact consequences of the master equation (Equation (32)) describing linear quantum field dynamics, both reversible and dissipative. One can include also nonlinear effects on the level of reduced states of the field by introducing the dependence on $\left(\hat{\rho },|\alpha \rangle \right)$ of the operators $\hat{h},{\hat{\gamma }}_{↑},{\hat{\gamma }}_{↓}$ and the measure μ(du) in the spirit of self-consistent Hartree–Fock approximation for bosons. Another generalization is needed to include the case of varying external conditions. This can be done by introducing also time-dependence into the operators $\hat{h},{\hat{\gamma }}_{↑},{\hat{\gamma }}_{↓}$, the measure μ(du) and the coherent external source |ξ〉. However, such extensions should be justified by the phenomenological arguments. One should remember that the Markovian approximation can be reconciled with time-dependent Hamiltonians only in the very particular situations such as slow, adiabatic modulation or fast periodic one (Floquet formalism) [13].

In the absence of coherent source, the equations become decoupled. Notice that, only in the absence of random source $\left({\hat{\gamma }}_{↑}=0\right)$ and random scattering (μ(du)=0) and under the assumption that the initial reduced state of the field is pure, i.e., $\hat{\rho }\left(0\right)=\left|\alpha \left(0\right)\rangle \langle \alpha \left(0\right)\right|$, it remains pure and satisfies classical field equation with damping and coherent source

(37) $$\frac{d}{dt}|\alpha \rangle =-\left(\frac{i}{\hbar }\hat{h}+\frac{1}{2}{\hat{\gamma }}_{↓}\right)|\alpha \rangle +|\xi \rangle .$$

Although this type of equations is quite frequently used, its applicability is limited to classical coherent sources, zero-temperature environment and the absence of random scattering.

## 6. Examples

To illustrate the presented formalism of reduced kinetic equations for reduced states of the field (Equations (35) and (36)), we consider two special cases: macroscopic field in thermal environment and linear polarization optics.

### 6.1. Thermal Environment

The reduced kinetic equations proposed above with possible nonlinear and time-dependent generalizations provide phenomenological tools to deal with macroscopic field interacting with an environment. There exist examples where special classes of the reduced kinetic equations can be derived from the underlying Hamiltonian models of the field interacting with the thermal bath and using appropriate approximations. The most popular approximation scheme combines Born, Markovian and secular ones and leads to the operators ${\hat{\gamma }}_{↑},{\hat{\gamma }}_{↓}$ diagonal in energy representation (we neglect also coherent source |ξ〉=0)

(38) $$\hat{\gamma_{↓}}=\sum_{k}\gamma_{↓}\left(k\right)|k\rangle \langle k|,\;\hat{\gamma_{↑}}=\sum_{k}\gamma_{↑}\left(k\right)|k\rangle \langle k|,$$

with the additional condition implied by the thermal character of the bath

(39) $$\gamma_{↑}\left(k\right)=e^{-(\hbar \omega_{k}/k_{B}T)}\gamma_{↓}\left(k\right).$$

The rates $\gamma_{↑}\left(k\right),\gamma_{↓}\left(k\right)$ can be computed using Fermi Golden Rule [14]. The random scattering term can also be derived using the alternative low density limit for the suitable Hamiltonian model of field—perturber elastic scattering. In this case, the measure μ(du) in Equations (35) and (36) is concentrated on unitaries commuting with $\hat{h}$. Under the above assumptions, one obtains the independent set of equations for the diagonal elements of a single-particle density matrix, $n_{k}=\langle k|{\hat{\rho }}_{1}|k\rangle$ (describing particle occupation numbers), and averaged field amplitudes $\alpha_{k}$

(40) $$\frac{d}{dt}n_{k}=-\left(\gamma_{↓}\left(k\right)-\gamma_{↑}\left(k\right)\right)n_{k}+\gamma_{↑}\left(k\right),$$

(41) $$\frac{d}{dt}\alpha_{k}=-\left(i\omega_{k}^{\prime }+\frac{1}{2}\left(\gamma_{↓}\left(k\right)-\gamma_{↑}\left(k\right)\right)+\gamma_{dec}\left(k\right)\right)\alpha_{k}$$

with the *decoherence rate*

(42) $$\gamma_{dec}\left(k\right)=ℜ\int \mu \left(du\right)\left(1-\langle k|\hat{u}|k\rangle \right)\geq 0.$$

Here, we assume that $ℑ\int \mu \left(du\right)\left(1-\langle k|\hat{u}|k\rangle \right)$ is absorbed into renormalized frequency $\omega_{k}^{\prime }$.

Equations (40) and (41) can be seen as the manifestation of *wave–particle duality* in the description of macroscopic field. The quantum-field feature of the system is hidden in the form of energy damping rates $\left(\gamma_{↓}\left(k\right)-\gamma_{↑}\left(k\right)\right)$ where the minus sign by the second term is a consequence of the *stimulated emission* related to bosonic character of field excitations (particles).

The quantum phenomenon of stimulated emission becomes particularly interesting for moving heat baths interacting with the macroscopic field. The case of rotating heat baths has been discussed in details in [15] where quantum master equations of the type in Equation (32) (with diagonal matrices $\left[{\Gamma_{↑(↓)}}^{kk^{\prime }}\right]$ and absent coherent sources and random scattering) were used. The only consequence of bath rotation is the modification of the relation in Equation (39) into

(43) $$\gamma_{↑}\left(k\right)=e^{-[\hbar \left(\omega_{k}-m\left(k\right)\Omega \right)/k_{B}T]}\gamma_{↓}\left(k\right).$$

where m(k) is a magnetic quantum number of the mode |k〉 and Ω is the angular frequency of rotation. The modes for which the *superradiance condition*

(44) $$\omega_{k}<m\left(k\right)\Omega$$

holds, possess a negative energy damping rate $\left(\gamma_{↓}\left(k\right)-\gamma_{↑}\left(k\right)\right)$, which means that the kinetic energy of rotating bath is pumped into these modes. Moreover, if the negative damping of the averaged mode amplitude $(\gamma_{↓}\left(k\right)-\gamma_{↑}\left(k\right))/2$ dominates over the decoherence rate $\gamma_{dec}\left(k\right)$, those amplitudes are amplified. This phenomenon is called *superradiance* and can be studied for various physical implementations: from Hawking radiation of rotating black holes to ocean wave generation by wind [15,16,17].

The quantum superradiance phenomenon is related to the classical phenomenon of shock waves. Namely, by introducing the radius *R* of the rotating bath, the linear velocity at its surface V=RΩ and the geometrical bound for the wave vector k≥m(k)/R, we can reformulate the superradiance condition in Equation (44) as

(45) $$V>v_{ph}\equiv \frac{\omega_{k}}{k}.$$

The above inequality is a typical condition for shock wave generation, i.e., the velocity of the source must be higher than the critical velocity given by the phase velocity $v_{ph}$ of the wave mode.

### 6.2. Polarization Optics Revisited

The method for reduced description of quantum field presented above can be applied to the polarization degrees of freedom in the case of linear optics devices. Namely, one can consider a light beam consisting of photons occupying the modes with a narrow band of frequencies around the central frequency $\omega^{0}$ and with a fixed spatial structure. Therefore, the reduced description in terms of reduced states of the field is given by the 2×2 positively defined Stokes matrix in Section 2, but now obtained from averaging over the full quantum state ${\hat{\rho }}_{F}$ of the light beam

(46) $$\hat{S}\equiv \left[S_{k\ell }\right],\;S_{k\ell }=\sum_{q}\mathrm{Tr}({\hat{\rho }}_{F}{\hat{a}}_{\ell q}^{†}{\hat{a}}_{kq}),\;k,\ell =1,2.$$

Here, *q* denotes the wave vectors of the beam modes, which are concentrated in the vicinity of the leading wave vector $q_{0}$ corresponding to the central frequency $\omega_{0}$ and defining the geometry of the beam. The transmission of the beam from the entrance to the exit of the linear optics device can be treated as the time evolution governed by the master equation of the type in Equation (35) with the absent coherent and incoherent sources (compare to the discussion of fiber optics in [18]). This evolution equation for the Stokes matrix reads

(47) $$\frac{d}{dt}\hat{S}=-i\left[\hat{\omega },\hat{S}\right]-\left\{{\hat{\gamma }}_{↓},{\hat{S}}_{1}\right\}+\int \mu \left(du\right)(\hat{u}\hat{S}{\hat{u}}^{†}-\hat{S}).$$

where:

(i) $\hat{\omega }$ is a Hermitian 2×2 matrix describing rotation of polarization vector;
(ii) ${\hat{\gamma }}_{↓}$ is a positive 2×2 matrix describing absorption of photons by the medium; and
(iii) $\hat{u}$ are 2×2 unitaries describing depolarization of light by random scattering with the positive weight μ(du).

From the discussion in the previous sections, it follows that the reduced description of a quantum field involves also the averaged field as an observable object. For polarization of a light beam, this is a two-dimensional complex vector $|\alpha \rangle =\left[\alpha_{1},\alpha_{2}\right]$, which is equivalent to the standard Jones vector with the normalization determined by the following definition

(48) $$|\alpha \rangle =\sum_{k=1}^{2}[\sum_{q}\mathrm{Tr}\left({\hat{\rho }}_{F}{\hat{a}}_{kq}\right)]|k\rangle .$$

The equation of motion for the averaged Jones vector is decoupled from Equation (47), but contains the same parameters

(49) $$\frac{d}{dt}|\alpha \rangle =-\left(i{\hat{\omega }}^{\prime }+\frac{1}{2}{\hat{\gamma }}_{↓}+{\hat{\gamma }}_{dec}\right)|\alpha \rangle$$

with ${\hat{\omega }}^{\prime }=\hat{\omega }+\hat{\delta }$, where

(50) $$\int \mu \left(du\right)\left(1-\hat{u}\right)=i\hat{\delta }+{\hat{\gamma }}_{dec},\;\hat{\delta }={\hat{\delta }}^{†},\;{\hat{\gamma }}_{dec}\geq 0.$$

Integrating the equation of motion (Equation (47)) between entry and exit times, we obtain a global dynamical map characterizing the influence of linear optics device on the polarization state. Because the first two terms on the right hand side of Equation (47) generate pure contracting completely positive maps and the third term generates bistochastic completely positive maps, the most general Mueller map satisfies

(51) $${\hat{S}}^{\prime }=\Phi \left(\hat{S}\right),\;\Phi \left(1\right)\leq 1,\;\Phi^{*}\left(1\right)\leq 1,$$

where $\Phi^{*}$ is a dual (Heisenberg picture) map. Such completely positive map can be called *doubly contracting*. In terms of the explicit representation

(52) $$\Phi \left(\hat{S}\right)=\sum_{\alpha }{\hat{V}}_{\alpha }\hat{S}{\hat{V}}_{\alpha }^{†},\;\Phi^{*}\left(\hat{M}\right)=\sum_{\alpha }{\hat{V}}_{\alpha }^{†}\hat{M}{\hat{V}}_{\alpha },$$

the conditions in Equation (51) can be written as

(53) $$\sum_{\alpha }{\hat{V}}_{\alpha }{\hat{V}}_{\alpha }^{†}\leq 1,\;\sum_{\alpha }{\hat{V}}_{\alpha }^{†}{\hat{V}}_{\alpha }\leq 1.$$

The Kraus decomposition in Equation (52) is not unique, but one can always choose at most four matrices ${\hat{V}}_{\alpha }$.

Similarly, the corresponding 2×2 matrix acting on Jones vectors is contracting

(54) $$|\alpha^{\prime }\rangle =\hat{V}|\alpha \rangle ,\;\hat{V}{\hat{V}}^{†}\leq 1.$$

In the most general case, the only condition which connects Φ and $\hat{V}$ is that for each pair of Stokes matrix $\hat{S}$ and Jones vector |α〉

(55) $$\hat{S}\geq |\alpha \rangle \langle \alpha |\;\mathrm{implies}\;\Phi \left(\hat{S}\right)\geq \hat{V}\left|\alpha \rangle \langle \alpha \right|{\hat{V}}^{†}.$$

The condition implies that the difference of two completely positive maps $\Phi -\hat{V}\cdot {\hat{V}}^{†}$ is positive.

Summarizing, in contrast to a general belief that Jones and Mueller calculi refer to physically different situations, we argue that the complete description of the polarization state of light beam needs a pair $\left(\hat{S},|\alpha \rangle \right)$ of Stokes matrix and averaged Jones vector satisfying $\hat{S}\geq \left|\alpha \rangle \langle \alpha \right|$. Equivalently, one can use Stokes parameters and explicit components of Jones vector in the given polarization basis $\left[s_{0},\vec{s};\alpha_{1},\alpha_{2}\right]$. The action of any linear optics device is described by a pair $\left(\Phi ,\hat{V}\right)$ of completely positive and doubly contracting Mueller map acting on 2×2 matrices and the Jones contracting 2×2 matrix such that the map $\Phi -\hat{V}\cdot {\hat{V}}^{†}$ is positive. The equivalent representation of $\left(\Phi ,\hat{V}\right)$ is given by a pair $\{\left[M_{\mu \nu }\right],\mu ,\nu =0,1,2,3;\left[V_{k\ell },k,l=1,2\right\}$ of Mueller and Jones matrices. We call such a set of pairs in both representations *Mueller–Jones maps*.

The set of Mueller–Jones maps form a semigroup with the composition of two elements

(56) $$\left(\Phi ,\hat{V}\right)\circ \left(\Phi^{\prime },\hat{V^{\prime }}\right)\equiv \left(\Phi \Phi^{\prime },\hat{V}\hat{V^{\prime }}\right),$$

which physically means a new optical device composed of two aligned ones.

Finally, we can settle the question of entropy for polarization of a light beam using the definition in Equation (31), which now takes the form

(57) $$S[\hat{S};|\alpha \rangle ]=k_{B}\mathrm{tr}(\left({\hat{S}}^{\alpha }+1\right)\log\left({\hat{S}}^{\alpha }+1\right)-{\hat{S}}^{\alpha }\log{\hat{S}}_{1}^{\alpha }),$$

with ${\hat{S}}^{\alpha }\equiv \hat{S}-\left|\alpha \rangle \langle \alpha \right|$.

## 7. Concluding Remarks

The mathematically consistent formalism of reduced description of quantum fields presented in this paper has a potentially wide range of applications. It is rather surprising that quantum features, in particular quantum statistics, have such an influence on the macroscopic behavior of wave-like excitations. Even for such macroscopic objects such as ocean waves generated by wind or magnetohydrodynamic waves in stellar atmospheres, the stimulated emission processes characteristic for bosons may lead to various macroscopic phenomena such as superradiance or creation of shock waves. The description in the form of two mathematical objects—averaged field and population numbers (diagonal elements of the single-particle density matrix)—can be seen as a macroscopic manifestation of particle–wave duality in the quantum world. Namely, for coherent sources, zero temperature environment and absent random scattering, the description in terms of wave equations with sources and pure damping is sufficient. When random/thermal effects prevail, the averaged field tends rapidly to zero and kinetic equations for (quasi)particle population numbers govern the evolution of the relevant observables. This is clearly visible for the case of moving sources (e.g., rotating heat bath). When the source velocity approaches the critical value incoherent production of (quasi)particles rapidly grows. At this moment, the classical wave description breaks down, which is interpreted as the creation of shock waves. However, the full macroscopic formalism of the reduced state of the field is valid. The averaged field part becomes irrelevant and the physical phenomena are described by the dynamics of the single-particle density matrix. Such “wave–particle transition” in the modeling of physical phenomena may explain the physical origin of singularities in purely classical theories such as hydrodynamics or general relativity. One can speculate that in general relativity when velocities of matter approach the speed of light, classical field description breaks down and the kinetic equations for graviton gas should be applicable.
